# Infected aneurysm of the thoracic aorta probably caused by *Bacillus cereus*: a case report

**DOI:** 10.1186/s12879-019-4602-2

**Published:** 2019-11-11

**Authors:** Tzu-Chi Wu, Ching-Chou Pai, Pin-Wen Huang, Chun-Bin Tung

**Affiliations:** 1 Department of Emergency Medicine, Show Chwan Memorial Hospital, 542, Sec 1 Chung-Shan Rd., Changhua, 500 Taiwan; 2 Division of Cardiovascular Surgery, Department of Surgery, Show Chwan Memorial Hospital, Changhua, Taiwan

**Keywords:** Infected aneurysm, *Bacillus cereus*, Biofilms

## Abstract

**Background:**

An infected aneurysm of the thoracic aorta is a rare clinical condition with significant morbidity and mortality. Patients with fast-growing aortic aneurysms show a high incidence of rupture. Gram-positive organisms, such as the *Staphylococ*cus and *Enterococcus* species, are the most common cause of infection.

**Case presentation:**

A 91-year-old man presented at our facility with high grade fever and tachypnea, which he had experienced for the previous two days. He had a history of end-stage renal disease and had been undergoing regular chest computed tomography (CT) follow-up for a left lower lung nodule. CT imaging with intravenous contrast media showed a thoracic aortic aneurysm with hemothorax. Rupture of the aneurysm was suspected. CT imaging performed a year ago showed a normal aorta. Blood samples showed a *Bacillus cereus* infection. The patient was successfully treated for a mycotic aortic aneurysm secondary to *Bacillus cereus* bacteremia.

**Conclusions:**

Here, we report a rare of an infected aneurysm of the thoracic aorta probably caused by *Bacillus cereus*. Although infected aneurysms have been described well before, an aneurysm infected with *Bacillus cereus* is rare. *Bacillus cereus*, a gram-positive spore-building bacterium, can produce biofilms, which attach to catheters. It has recently emerged as a new organism that can cause serious infection.

## Background

*Bacillus cereus* is a gram-positive rod-shaped bacteria found in fresh water, soil, and hospital surroundings. It is associated mainly with food poisoning, but it can cause severe infection, such as osteomyelitis, meningitis, and endocarditis, particularly in immunosuppressed individuals [1]. An infected aneurysm is a rare and life-threatening clinical condition that is associated with significant morbidity and mortality. Here, we report a rare case of infectious thoracic aneurysm probably caused by *Bacillus cereus*. To the best of our knowledge, this is the first report of an infected aortic aneurysm probably caused by *Bacillus cereus*.

## Case presentation

A 91-year-old male presented with high grade fever, chills, and tachypnea. He had been experiencing these symptoms for two days prior to presentation. He had a history of end-stage renal disease and for the previous 8 months had a PermCath emplacement in the right internal jugular vein for regular hemodialysis treatment. For the past year he had also been receiving regular computed tomography (CT) follow up for a left lower lung nodule. He denied obvious chest or abdominal pain. On physical examination his blood pressure was 127/87 mmHg, pulse rate 112/min, respiration rate 20/min, and body temperature 39.6 °C. A cardiopulmonary examination showed coarse breathing sounds over the left lung and regular heart beat without jugular venous distention. Abdominal and neurological examinations showed unremarkable findings. Femoral, popliteal, and dorsalis pedis pulses were equal and palpable bilaterally.

A laboratory evaluation revealed a white blood cell count of 20,050/uL (reference range, 4000–10,000/uL) with 73% segmented neutrophils, hemoglobin 11.7 mg/dL (reference range, 12–14 mg/dL), creatinine 6.65 mg/dL, high-sensitivity C-reactive protein (CRP) 30.45 mg/dL (reference range, < 0.3 mg/dL), blood pH 7.46, HCO3 20.9 mmol/L, base excess − 2.1 mmol/L, and pCO2 30.1 mmHg.

**Fig. 1:**
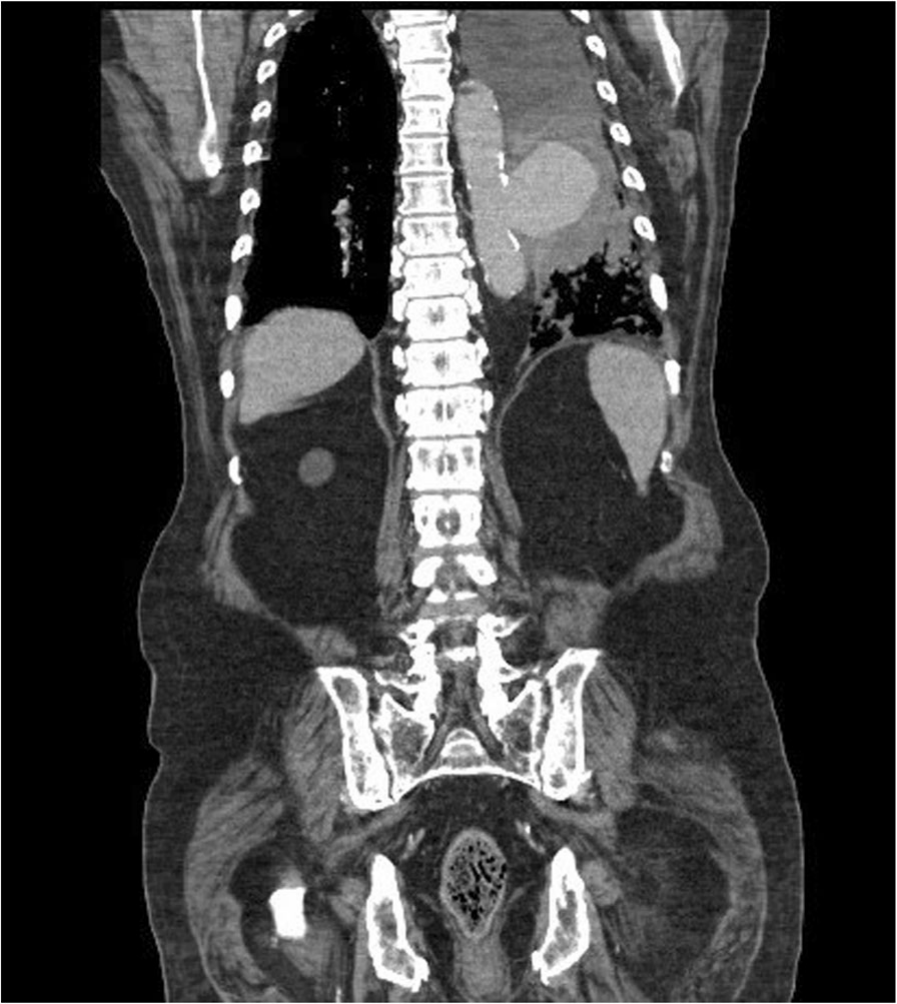
Computed tomography scans with intravenous contrast media show a thoracic aortic aneurysm with hemothorax

**Fig. 2:**
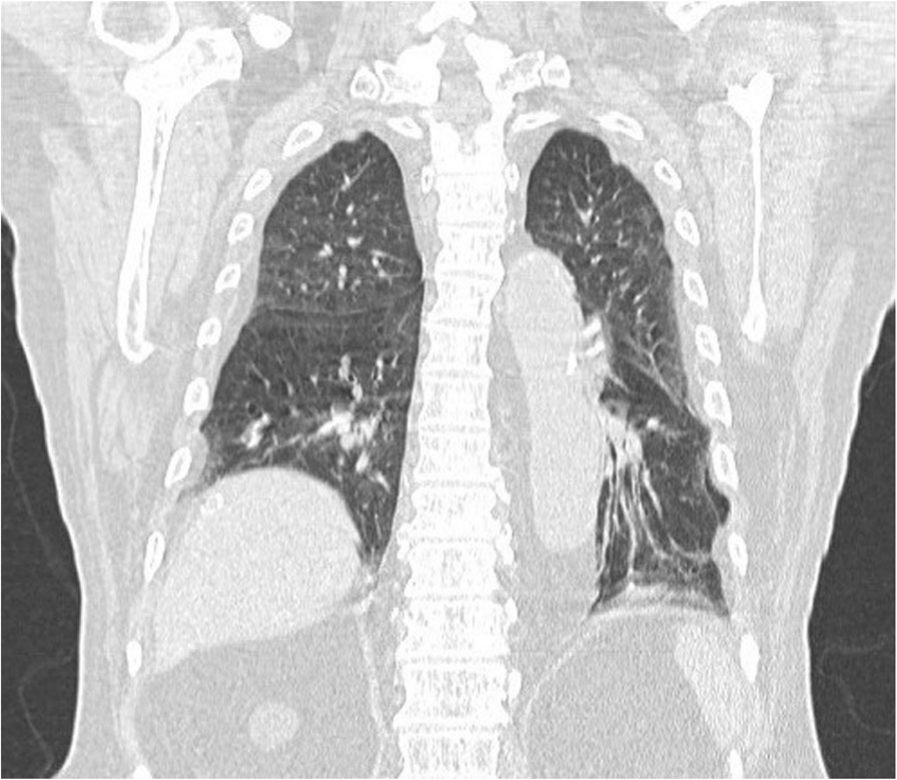
A computed tomography scan from a year prior to presentation shows a normal aorta

Chest X-ray showed widening of the mediastinum with left costophrenic angle blunting. Due to detection of the serious infection without a focused infection site and the chest X-ray findings, further imaging was prescribed. CT imaging with intravenous contrast media showed a thoracic aortic aneurysm (5.7 cm × 7 cm × 9 cm) at the T8 level with atelectasis of the left lung and hemothorax (Fig. 1). A CT scan for a lung nodule from a year prior to presentation showed a normal aorta (Fig. 2). The CT results combined with clinical symptoms of high fever and leukocytosis with elevated CRP, led us to suspect an infectious thoracic aneurysm. The patient was transferred to ICU. Intravenous vancomycin 1 g 3 times per week and intravenous ceftriaxone 1 g every 12 h were started empirically. The patient developed fulminant septic shock and acute respiratory failure. An emergency thoracic endovascular aortic repair (TEVAR) for the thoracic aortic aneurysm was performed. After TEVAR and antibiotic administration, the patient’s septic shock improved and his clinical condition stabilized.

**Fig. 3:**
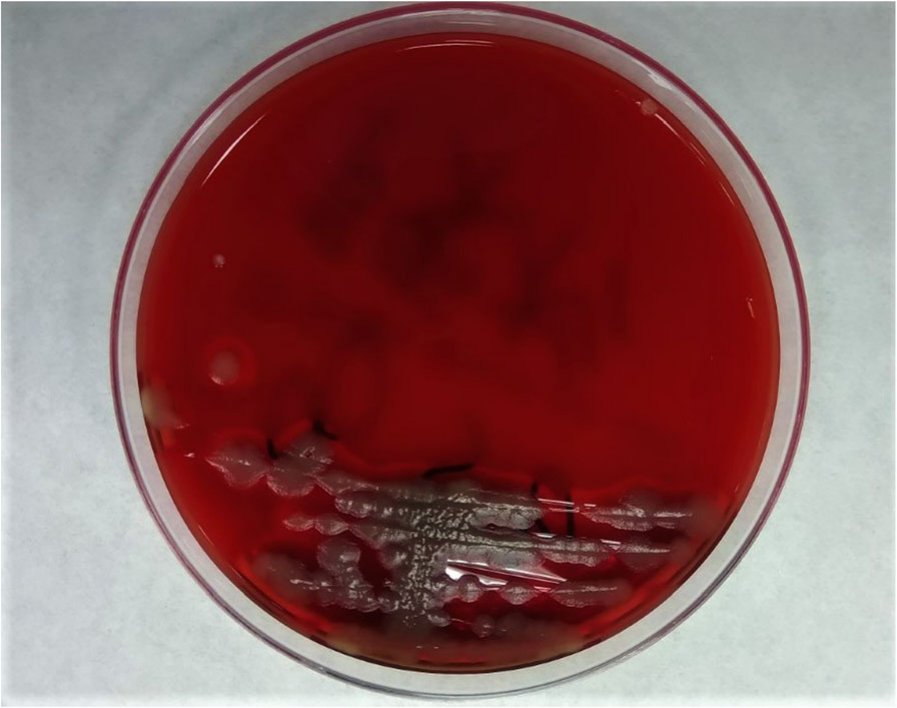
Gray colonies growing on sheep blood agar indicate beta-hemolytic bacteria

**Fig. 4:**
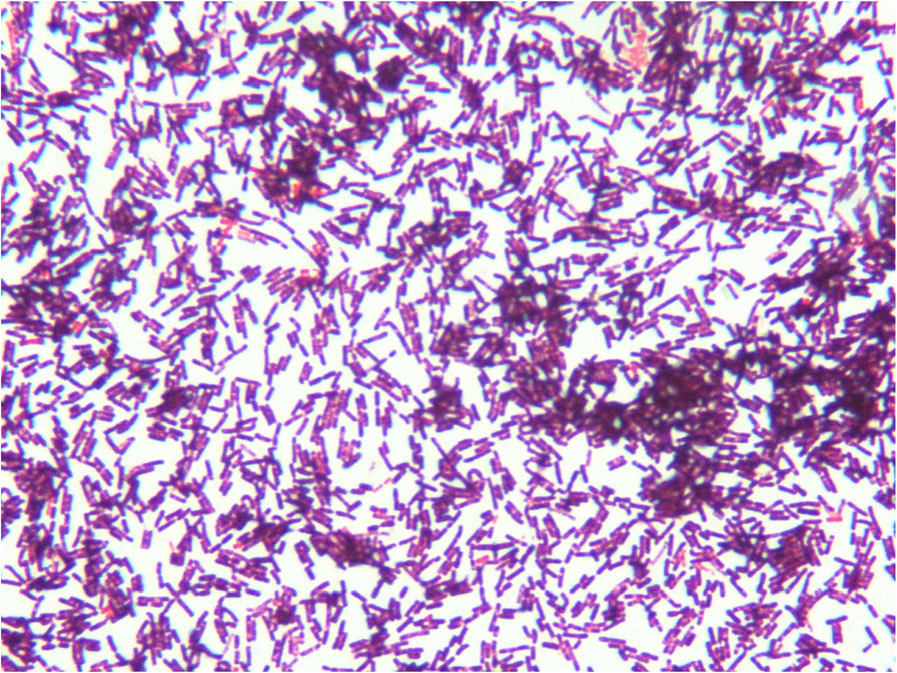
Blood culture shows gram-positive, rod-shaped bacteria

Post admission day 3 and day 5, *Bacillus cereus* was isolated in two sets of aerobic culture obtained from the peripheral vein. A third culture obtained from the PermCath at admission showed *Bacillus cereus* with a 24-h shorter positive time than those from the peripheral vein. Gray colonies with irregular perimeters were seen growing on sheep blood agar indicating beta-hemolytic bacteria (Fig. 3). Gram staining revealed gram-positive *Bacillus* species (Fig. 4). *Bacillus cereus* was isolated using the BD Phoenix 100 system. However, no bacteria grew in an anaerobic culture. Post antibiotic administration and during follow up on days 2, 3, and 4, a PermCath tip sample and three blood samples were negative for both anaerobic and aerobic cultures.

After detection of *Bacillus cereus* bacteremia, the patient was administered intravenous vancomycin for 6 weeks followed by fluoroquinolones oral form for 4 weeks and the PermCath was removed. Ultrasound imaging of the heart showed no pericardial effusion and no obvious vegetation. After the treatment, the patient remained asymptomatic for 6 months, without signs of relapse of infection.

## Discussion and conclusions

Here, we report a rare of an infected aneurysm of the thoracic aorta probably caused by *Bacillus cereus* that was successfully treated by surgical and antibiotic interventions. *Bacillus cereus* typically presents as a gastrointestinal infection, but many cases have been reported recently that indicate it can cause life-threatening and systemic infections, such as osteomyelitis, necrotizing fasciitis, endophthalmitis, meningitis, and endocarditis, particularly in neonates, immunocompromised individuals, and intravenous drug abusers [1]. Therefore, *Bacillus cereus* bacteremia is a growing concern as a cause of invasive infection. However, cardiovascular complications of *Bacillus cereus* infection probably resulting in infected aneurysms have not r been reported previously.

The clinical isolate of *Bacillus cereus* is usually regarded as a contaminant, but it is still one of the pathogens causing nosocomial bloodstream infections [1]. The possible infectious routes may include bacterial contamination through the skin, venous catheter, or healthcare providers. In a retrospective study of 29 *Bacillus cereus* bloodstream infection cases, the main etiology was venous catheter-related (69%) [1]. In our case, an arteriovenous fistula was not made for hemodialysis due to the patient’s old age. The PermCath was used for hemodialysis for about 8 months without infection. However, at the present presentation, laboratory analysis showed that the same bacteria were present in three blood cultures (two from the peripheral vein obtained at an interval of 8 h and one from the PermCath) which meets the criteria for catheter-related bloodstream infection. The gram-positive time of the culture from the PermCath was shorter than those from the peripheral vein by about 24 h [2]. Following admission and post antibiotic administration, a culture from the tip of the PermCath and three blood cultures from post admission days 3, 4, and 5 were negative. The BD Phoenix™ system was used to identify the bacterial isolates. Antimicrobial susceptibility is determined by the antimicrobial breakpoints of *Bacillus cereus* in the Clinical & Laboratory Standards Institute (CLSI) guidelines [1]. However, we did not conduct susceptibility tests because our laboratory could not meet the *Bacillus cereus* testing criteria provided in the guidelines.

Generally, the central-line-associated bloodstream infection rate in intensive care units is estimated to be 0.8 per 1000 central line days. The common pathogens causing central-line-associated bloodstream infections include *Staphylococci*, *Enterococci*, *Staphylococcus aureus* and *Klebsiella* [3]. *Bacillus* species-related infections are rare in central-line-associated bloodstream infections and some reports suggest that biofilm-forming strains of the *Bacillus* species can cause nosocomial bacteremia by catheter infection [4]. The biofilms are an important component of bacterial survival which enables them to form communities, adhere to surfaces, and resist antibiotics [5–7]. In addition, the presence of indwelling medical devices increases the risk of biofilm formation [5].

An infected thoracic aortic aneurysm is a rare clinical condition and accounts for 1–3% of all aneurysms. Patients with an infected thoracic aortic aneurysm are associated with high incidences of rupture due to the fast growth of aortic aneurysms [8]. More than 60% of the pathogens causing aortic infection are gram-positive bacteria such as the *Staphylococcal*, *Enterococcus*, and *Streptococcus pneumoniae* species [9].

There are four main mechanisms that may describe the pathogenesis of infected aortic aneurysms. They include septic emboli, bacteremic seeding, direct bacterial inoculation at the time of trauma and contiguous infection. An intima with atherosclerosis allows bacteria to enter the aortic wall and is the most common pathogenesis of an infected aortic aneurysm, especially in elderly patients [9]. There were no perioperative cultures and pathologies to confirm the pathogenesis because endovascular surgery had been performed. An infected aortic aneurysm was suspected due to the clinical symptoms, laboratory examinations and radiological findings, including a mushroom-like appearance and fast-growing aorta aneurysm which grew from normal to nearly 7 cm within a year. We hypothesized that *Bacillus cereus* bacteremia seeding of the atherosclerotic plaque led to arterial wall infection.

In general, 4–6 weeks of vancomycin is optimal for treatment of *Bacillus cereus* bacteremia. In a retrospective study of 29 *Bacillus cereus* bloodstream infection cases, all isolates showed good susceptibility to vancomycin, MIC ≤2 (CLSI’s breakpoint for vancomycin is ≤4) [1]. However, most *Bacillus cereus* are resistant to penicillin and cephalosporins due to the production of β-lactamase. Other antimicrobial options, including fluoroquinolones, carbapenem, clindamycin, daptomycin and linezolid, may be effective only if the *Bacillus cereus* isolate is susceptible. This is because high resistance rates are still noted in some studies–approximately 10.3% for levofloxacin and 65.5% for clindamycin [1, 9, 10]. In our case, the patient was treated with vancomycin for 6 weeks followed by fluoroquinolones oral use. His clinical condition improved and a PermCath tip culture (3 days post antibiotic treatment) and three sets of blood culture in serial were negative. Generally, the antibiotic treatment can be extended for 6 to 12 weeks after surgical excision and even longer if a patient’s immunosuppressed condition is noted. Some authors suggest that life-long parenteral antibiotics may be considered if a patient has active bacteremia [9, 10].

Endovascular aortic repair may be an alternative to antibiotics for treating infected aortic aneurysms. However, the risk of recurrence of infection may be high due to embedded infection. In a systematic review of 48 cases of mycotic aortic aneurysm from 22 reports indicates that endovascular aortic repair should be considered as a method to achieve hemodynamic stability when patients present with rupture or shock [10]. In patients with comorbidities, emergency endovascular stenting may be indicated to treat ruptured infected aortic aneurysms when open repair is not possible [8, 11].

*Bacillus cereus* can produce biofilms, which can attach to catheters and lead to bacteremia. *Bacillus cereus* bacteremia is a growing concern as a cause of invasive infection especially in immunocompromised individuals.

## Data Availability

The datasets analyzed during the current case report are available from the corresponding author on reasonable request.

## Abbreviations

CLSI: Clinical & Laboratory Standards Institute
CT: computed tomography
TEVAR: Thoracic Endovascular Aortic Repair
